# Intermittent Hypoxia and Hypercapnia Reproducibly Change the Gut Microbiome and Metabolome across Rodent Model Systems

**DOI:** 10.1128/mSystems.00058-19

**Published:** 2019-04-30

**Authors:** Anupriya Tripathi, Zhenjiang Zech Xu, Jin Xue, Orit Poulsen, Antonio Gonzalez, Gregory Humphrey, Michael J. Meehan, Alexey V. Melnik, Gail Ackermann, Dan Zhou, Atul Malhotra, Gabriel G. Haddad, Pieter C. Dorrestein, Rob Knight

**Affiliations:** a Division of Biological Sciences, University of California—San Diego, San Diego, California, USA; b Department of Pediatrics, University of California—San Diego, San Diego, California, USA; c Skaggs School of Pharmacy and Pharmaceutical Sciences, University of California—San Diego, San Diego, California, USA; d State Key Laboratory of Food Science and Technology, Nanchang University, Nanchang, China; e Division of Pulmonary, Critical Care and Sleep Medicine, University of California—San Diego, San Diego, California, USA; f Department of Neuroscience, University of California—San Diego, San Diego, California, USA; g Collaborative Mass Spectrometry Innovation Center, University of California—San Diego, San Diego, California, USA; h Center for Microbiome Innovation, University of California—San Diego, San Diego, California, USA; i Department of Computer Science and Engineering, University of California—San Diego, San Diego, California, USA; j Rady Children’s Hospital—San Diego, San Diego, California, USA; California Department of Water Resources

**Keywords:** cardiovascular, machine learning, metabolism, microbiome, sleep apnea

## Abstract

Studying perturbations in the gut ecosystem using animal models of disease continues to provide valuable insights into the role of the microbiome in various pathological conditions. However, understanding whether these changes are consistent across animal models of different genetic backgrounds, and hence potentially translatable to human populations, remains a major unmet challenge in the field. Nonetheless, in relatively limited cases have the same interventions been studied in two animal models in the same laboratory. Moreover, such studies typically examine a single data layer and time point. Here, we show the power of utilizing time series microbiome (16S rRNA amplicon profiling) and metabolome (untargeted liquid chromatography-tandem mass spectrometry [LC-MS/MS]) data to relate two different mouse models of atherosclerosis—ApoE^−/−^ (*n* = 24) and Ldlr^−/−^ (*n* = 16)—that are exposed to intermittent hypoxia and hypercapnia (IHH) longitudinally (for 10 and 6 weeks, respectively) to model chronic obstructive sleep apnea. Using random forest classifiers trained on each data layer, we show excellent accuracy in predicting IHH exposure within ApoE^−/−^ and Ldlr^−/−^ knockout models and in cross-applying predictive features found in one animal model to the other. The key microbes and metabolites that reproducibly predicted IHH exposure included bacterial species from the families *Mogibacteriaceae*, *Clostridiaceae*, bile acids, and fatty acids, providing a refined set of biomarkers associated with IHH. The results highlight that time series multiomics data can be used to relate different animal models of disease using supervised machine learning techniques and can provide a pathway toward identifying robust microbiome and metabolome features that underpin translation from animal models to human disease.

**IMPORTANCE** Reproducibility of microbiome research is a major topic of contemporary interest. Although it is often possible to distinguish individuals with specific diseases within a study, the differences are often inconsistent across cohorts, often due to systematic variation in analytical conditions. Here we study the same intervention in two different mouse models of cardiovascular disease (atherosclerosis) by profiling the microbiome and metabolome in stool specimens over time. We demonstrate that shared microbial and metabolic changes are involved in both models with the intervention. We then introduce a pipeline for finding similar results in other studies. This work will help find common features identified across different model systems that are most likely to apply in humans.

## Abstract (precis)

Reproducibility of microbiome research is a major topic of contemporary interest. Although it is often possible to distinguish individuals with specific diseases within a study, the differences are often inconsistent across cohorts, often due to systematic variation in analytical conditions. Here we study the same intervention in two different mouse models of cardiovascular disease (atherosclerosis) by profiling the microbiome and metabolome in stool specimens over time. We demonstrate that shared microbial and metabolic changes are involved in both models with the intervention. We then introduce a pipeline for finding similar results in other studies. This work will help find common features identified across different model systems that are most likely to apply in humans.

## INTRODUCTION

Obstructive sleep apnea (OSA) is a common sleep disorder marked by obstructed breathing due to episodic upper airway collapse. Chronic OSA is associated with adverse cardio-metabolic outcomes such as atherosclerosis (1); however, potential causal pathways remain elusive. We previously modeled human OSA and its cardiovascular consequences in Ldlr knockout (Ldlr^−/−^ [atherosclerosis model]) mice by exposing individuals to intermittent hypoxia and hypercapnia (IHH), a hallmark of OSA (2). IHH is a clinically important exposure because it markedly promotes atherosclerotic lesions in the pulmonary arteries and aorta not only in Ldlr^−/−^ mice but also in ApoE knockout (ApoE^−/−^) mice, another widely used atherosclerosis model (3, 4), thereby mimicking the adverse cardiovascular changes that occur in OSA patients (5). In Ldlr^−/−^ mice, we reported significant shifts in the bacterial and chemical composition of the gut on IHH exposure. The key chemical alterations included changes in microbe-dependent metabolites such as gut-derived estrogen-like molecules (phytoestrogens) and bile acids. These observations revealed an unrecognized link between IHH and gut microbes, thereby holding immense potential for translation in OSA patients. However, a key challenge in microbiome research is understanding if different animal models or animal models and human subjects are characterized by common changes in the microbiome and metabolome (6, 7). As a first step, finding reproducible alterations across multiple animal models would provide confidence in the generalizability of the findings and accrue evidence for clinical relevance.

Here, we use machine learning predictive models to address the reproducibility of the perturbations associated with IHH exposure in the gut ecosystem using both Ldlr^−/−^ and ApoE^−/−^ mouse models (see Fig. S1 in the supplemental material). To model OSA and its cardiovascular conditions, all mice were exposed to either IHH (treatment group) or air (control group) and fed a high-fat diet (HFD) (3, 4). Individuals were studied longitudinally for 6 weeks (Ldlr^−/−^) or 10 weeks (ApoE^−/−^) to understand the impact of prolonged IHH exposure (analogous to chronic OSA in humans). Furthermore, multiple cages per treatment group were used following study design recommendations (8). Starting with 10 weeks of age (baseline), fecal pellets were collected twice every week and profiled for the microbiome and metabolome using 16S rRNA amplicon sequencing and liquid chromatography-tandem mass spectrometry (LC-MS/MS)-based untargeted mass spectrometry, respectively. These data layers were processed per recommended practices (9) to obtain relative abundances of microbial and molecular species per sample for all downstream analyses (see Materials and Methods).

10.1128/mSystems.00058-19.1 FIG S1 Schematic illustration of treatment paradigm and sample collection. Groups of 8-week-old male Ldlr^−/−^ or ApoE^−/−^ mice were transferred to the treatment room for 2 weeks of acclimatization with room air and regular chow food. At 10 weeks of age, mice were switched to high-fat diet (HFD) and treated with or without intermittent hypoxia and hypercapnia (IHH). The IHH treatment group received 10 h/day IHH in the light cycle for 6 weeks in Ldlr^−/−^ mice or for 10 weeks in ApoE^−/−^ mice. The blue line is the O_2_ set point, and the green line is the actual level of O_2_. The red line is the CO_2_ set point, and light blue is the actual level of CO_2_. The control groups remained in room air for the same period. All the mice were reared in the same animal facility and thus have the same microbial exposure. Fecal pellets were collected at baseline and twice per week thereafter and were used for microbiome and metabolome analyses. All the time points were analyzed except for the metabolome of ApoE^−/−^ mice (for whom only fecal samples at the age of 10, 12, 14.5, 17, and 19.5 weeks were analyzed). For Ldlr^−/−^ mice, for IHH, mouse no. 17 to 20 and 21 to 24 were kept in cage numbers 5 and 6, respectively, and for air, mouse no. 25 to 28 and 29 to 32 were kept in cage no. 7 and 8, respectively. For ApoE^−/−^ mice, for IHH, mouse no. 97 to 100, 101 to 104, and 105 to 108 were kept in cage numbers A18, A19, and A20, respectively, and for air, mouse no. 109 to 112, 113 to 116, and 117 to 120 were kept in cage no. A21, A22, and A23, respectively. Comprehensive sample metadata are available publicly (see “Data availability” in the article). Download FIG S1, TIF file, 1.2 MB. Copyright © 2019 Tripathi et al. 2019 Tripathi et al. This content is distributed under the terms of the Creative Commons Attribution 4.0 International license.

Predictive models that classify microbiome and metabolome responses to interventions have proven extremely useful in disease diagnosis and biomarker discovery (10, 11). Yet, these have been surprisingly hard to generalize across populations or model systems (12–14). In this work, we use random forest (RF) classification to investigate the cross-applicability of our previous findings in Ldlr^−/−^ mice (2) to ApoE^−/−^ mice and vice versa. RF is an ensemble machine learning algorithm that fits many decision trees on random subsamples of original data and then aggregates the results of each decision tree to improve the prediction accuracy (15). The level of accuracy is often expressed using the area under the curve (AUC) of true-positive versus false-positive rates, known as a receiver operating characteristic (ROC). RF has consistently been reported to perform well in high-dimensional data sets (i.e., data sets with many features [microbial reads or metabolites] such as ours), making it our algorithm of choice for this work (16–18). We had previously shown that machine learning classifiers trained on inflammatory bowel disease (IBD) cases and healthy controls in humans can distinguish between IBD cases and controls in dogs using cross-sectional microbiome data (19). To our knowledge, however, this type of cross-model classification task has not been performed with metabolomics data or with data collected longitudinally.

## RESULTS

### Unsupervised comparison of the gut microbiome and metabolome in ApoE^−/−^ and Ldlr^−/−^ mouse models.

**FIG 1:**
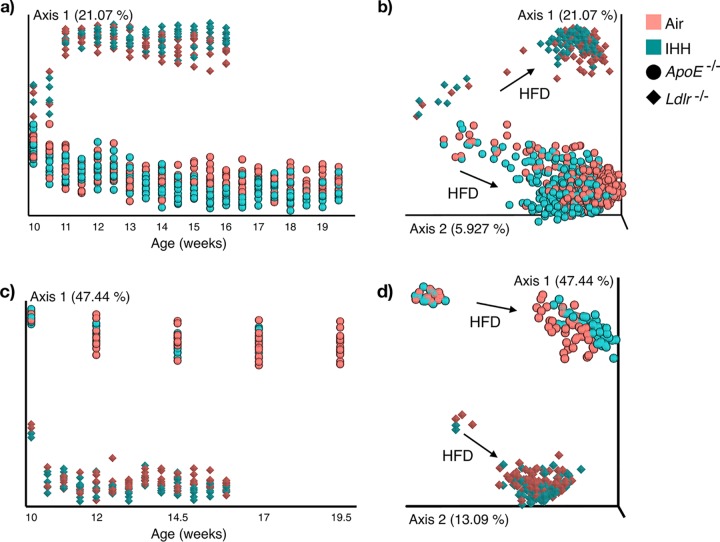
Principal-coordinate analysis (PCoA) of the gut microbiome and metabolome in *ApoE*^−/−^ and *Ldlr*^−/−^ mouse models. (a and b) PCoA of the microbiome (16S rRNA sequencing) data using unweighted UniFrac distances. (c and d) PCoA of the metabolome (untargeted LC-MS/MS) data using Bray-Curtis distances. The ordination is visualized along the duration of treatment (starting at 10 weeks of age, with an interval of 0.5 week). Axis 1, principal coordinate 1; IHH, intermittent hypoxia and hypercapnia; HFD, high-fat diet.

First, we performed principal-coordinate analysis (PCoA) to get a visual overview of the characteristic microbiome and metabolome of the two animal models. PCoA is an unsupervised method routinely used to explore major factors that drive the clustering of data points in high-dimensional data sets by projecting the samples in a reduced-dimensional space (as a two-dimensional [2D] or 3D graph) (20). Figure 1 displays the PCoA results plotted along time to visualize the dynamics of diet and IHH-associated changes in the gut ecosystem. This analysis shows that the Ldlr^−/−^ and ApoE^−/−^ mice in our study have very distinct gut microbial (Fig. 1a and b) and chemical (Fig. 1c and d) signatures, which are captured by the first principal axis (axis 1) in both data layers. These plots also capture a rapid shift in the baseline gut microbial and chemical compositions in response to HFD, which has also been reported previously (21, 22). We performed PCoA without baseline samples to better visualize the impact of IHH exposure alone (see Fig. S2 in the supplemental material). We observed that despite underlying differences in the two genotypes, axis 2 consistently captured IHH-induced shifts in both the gut microbiome and metabolome, highlighting common shifts in the gut ecosystem due to IHH exposure.

10.1128/mSystems.00058-19.2 FIG S2 Principal-coordinate analysis (PCoA) of the gut microbiome and metabolome in *ApoE*^−/−^ and *Ldlr*^−/−^ mouse models without baseline samples. (a) PCoA of the gut microbiome using unweighted UniFrac after removal of samples collected at 10 and 10.5 weeks of age. (b) Similar PCoA for gut metabolome using Bray-Curtis distances. Baseline samples were removed from this plot to better visualize the impact of IHH exposure alone. Axis 1 explains the variability due to the animal models, and axis 2 consistently captures the IHH-associated changes in the microbiome and metabolome in both models. Axis 1, principal coordinate 1; IHH, intermittent hypoxia and hypercapnia. Download FIG S2, TIF file, 0.6 MB. Copyright © 2019 Tripathi et al. 2019 Tripathi et al. This content is distributed under the terms of the Creative Commons Attribution 4.0 International license.

When comparing the overall sharedness of microbial features, out of 635 unique 16S Deblur processed sequences (23) in the Ldlr^−/−^ gut microbiome (and 582 in the ApoE^−/−^ gut microbiome), the two animal models only shared 248 unique sequences. The chemical space was also distinct, with the two models sharing only 137 out of 267 and 374 metabolomic features in Ldlr^−/−^ and ApoE^−/−^ mice, respectively (see methods). Interestingly, Ldlr^−/−^ and ApoE^−/−^ mice have more similar microbiomes (by permutational analysis of variance [PERMANOVA] [24], pseudo-*F*, 13.2605, and *P* < 0.001) at baseline (10 weeks of age [i.e., before the HFD-induced shift is observed]) compared to later time points (pseudo-*F*, 19.9059 at 12 weeks of age). We note a similar divergence in the gut metabolome (pseudo-*F*, 46.9112 and 66.1165 at baseline and 12 weeks of age, respectively) of the two animal models as well. Together, these results suggest a differential impact of high-fat diet on the gut ecosystem of the Ldlr^−/−^ and ApoE^−/−^ mice which makes the mouse models more distinct over time.

It is important to note that the two animal models are temporally separated for sample collection and data acquisition (for both 16S rRNA sequencing and LC-MS/MS), which likely contributes to the strong distinction between the models observed here. We quantified the effects of covariates such as genotypes (or processing batches), age of individuals, housing conditions, and individual variability on the microbiome and metabolome composition by performing effect size analysis on our data set (see the “Effect size analyses” section in Materials and Methods). While the largest effect on the microbial and chemical composition was linked to the mouse model, the type of exposure (IHH or air) impacted each data layer within both models significantly. Moreover, the effect sizes varied based on the animal model, highlighting the distinctive characteristics of the gut ecosystem in the two models (see Table S1 in the supplemental material).

10.1128/mSystems.00058-19.5 TABLE S1 Effect size of covariates for the microbiome (a to c) and metabolome (d to f) data set using mixed directional FDR (60). Download Table S1, DOCX file, 0.1 MB. Copyright © 2019 Tripathi et al. 2019 Tripathi et al. This content is distributed under the terms of the Creative Commons Attribution 4.0 International license.

### Gut microbiome- and metabolome-based prediction of IHH exposure within and across animal models.

Our unsupervised analysis showed that the gut ecosystems of ApoE^−/−^ and Ldlr^−/−^ mice, despite being inherently distinct, consistently shift in response to IHH exposure (Fig. S2). We applied supervised machine learning in order to capture the consistent shifts associated with IHH exposure in both animal models. Specifically, we built RF classifiers using IHH-associated microbial and chemical composition in ApoE^−/−^ and tested their performance in predicting IHH exposure in Ldlr^−/−^ mice and vice versa. This informed us if the changes we observe in one model are consistent with the other, which would make the findings more relevant for translation in OSA patients.

**FIG 2:**
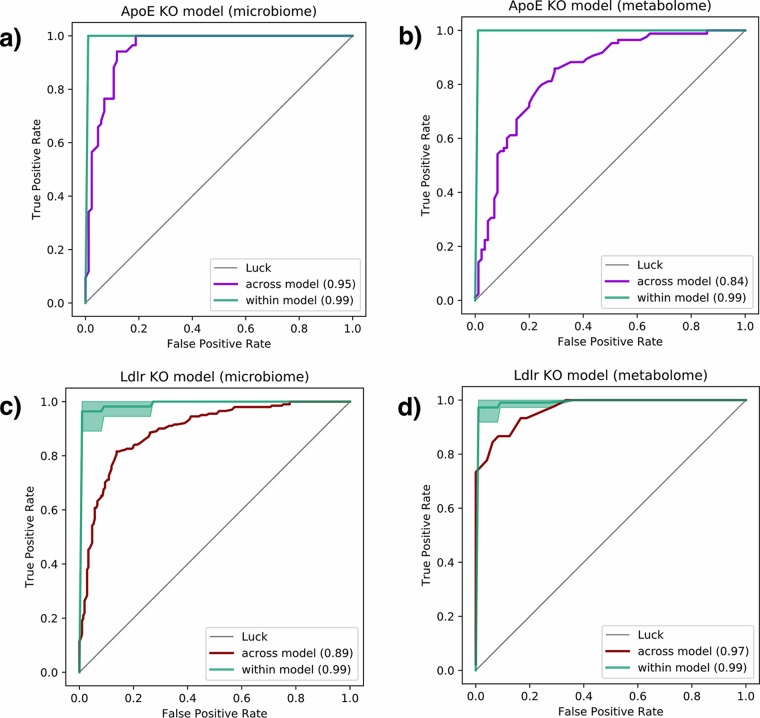
Receiver operating characteristic (ROC) curves evaluating ability to predict exposure to IHH using the random forest model. Green curves represent classification accuracy within each mouse model. Purple ROC curves correspond to a model trained using gut microbiome (a) and metabolome (b) data from the *ApoE*^−/−^ mouse model to predict IHH exposure in *Ldlr*^−/−^ mice. Red curves show the same for microbiome (c) and metabolome (d) data from *Ldlr*^−/−^ mice tested on *ApoE*^−/−^ mice. IHH, intermittent hypoxia and hypercapnia.

To examine the predictive potential of microbiome data, we trained RF classifiers on relative abundances of 16S tag sequences shared between the two mouse models (443 features after removing low-prevalence sequences [see Materials and Methods]). Within each animal model, the classifiers yielded nearly perfect prediction (99% area under the ROC curve [AUC]) of IHH exposure (Fig. 2). We then predicted the same in Ldlr^−/−^ using RF trained on microbiome signature in ApoE^−/−^ (Fig. 2a) and vice versa (Fig. 2c), still achieving very high cross-model prediction accuracies (95% and 89% AUC, respectively). Similarly, we used metabolomics data for training RF models on relative abundance of MS1 spectral ions (377 features after removing low-prevalence ions [see Materials and Methods]). Metabolome-based RF classifiers also predicted IHH exposure within animal models accurately (99% AUC) and maintained impressive cross-model prediction accuracies (97% when trained on Ldlr^−/−^ mice and tested on ApoE^−/−^ mice [Fig. 2b] and 84% vice versa [Fig. 2d]). Together, these analyses suggest that IHH exposure alters both the gut microbial and chemical compositions distinguishably in each animal model. Moreover, the changes induced by IHH exposure in the gut are largely consistent across Ldlr^−/−^ and ApoE^−/−^ models, despite the underlying differences between the two genotypes (Fig. 1; Fig. S2). It is worth noting that we hugely benefited from our longitudinal sample collection scheme as we had more data points available for learning, despite the limited number of animals (i.e., *n* = 8 for Ldlr^−/−^ and *n* = 12 for ApoE^−/−^) per group. We accounted for the longitudinal samples from the same individual in our analyses by ensuring that observations for each individual appeared in either the training or validation data set but not both. This prevented overoptimistic cross-validation accuracy scores as a result of the model overfitting to the characteristics of the individual itself rather than the treatment. (The relatively lower accuracy of ApoE^−/−^-based metabolomics RF model can be attributed to fewer samples compared to the Ldlr^−/−^ model [Fig. S1].)

### Longitudinal dynamics of IHH-associated changes in the gut ecosystem.

Next, we used these longitudinal data sets to learn how the duration of IHH exposure impacts the gut microbiome and metabolome over time and if this is consistent across the mouse models. The goal was to compare the dynamics of changes in the gut ecosystem with chronic IHH exposure in the ApoE^−/−^ and Ldlr^−/−^ mice. We tested this by assessing the capability of the RF classifier to distinguish IHH samples from the control at each time point. In ApoE^−/−^ mice, the classification AUC using gut microbiome data is high (constantly 1) at each time point starting at 11 weeks of age. The microbiome in Ldlr^−/−^ mice, however, appears more predictive only at later time points, with its classification AUC improving from 0.71 at week 11 to more than 0.99 beyond week 14. We also observed a similar lag in gut metabolome changes in Ldlr^−/−^ compared to ApoE^−/−^ animals (see Table S2 in the supplemental material). Importantly, this is concordant with our previous finding that the atherosclerotic lesions evolved slowly and mildly in Ldlr^−/−^ mice compared to ApoE^−/−^ mice (4). Therefore, observing this trend in both omics layers provides supporting evidence that the atherosclerosis phenotype in these animals is linked to perturbations in their gut ecosystem. Moreover, the gut microbiome and metabolome changes occur quickly after IHH exposure, before atherosclerotic lesions were observed, which was reported to be 4 weeks for ApoE^−/−^ mice and 6 weeks for Ldlr^−/−^ mice post-IHH exposure (4).

10.1128/mSystems.00058-19.6 TABLE S2 Prediction of intermittent hypoxia and hypercapnia for each time point using (a) microbiome and (b) metabolome data in *ApoE*^−/−^ and *Ldlr*^−/−^ animals. Download Table S2, DOCX file, 0.1 MB. Copyright © 2019 Tripathi et al. 2019 Tripathi et al. This content is distributed under the terms of the Creative Commons Attribution 4.0 International license.

### Reproducible biomarkers of IHH exposure.

**FIG 3:**
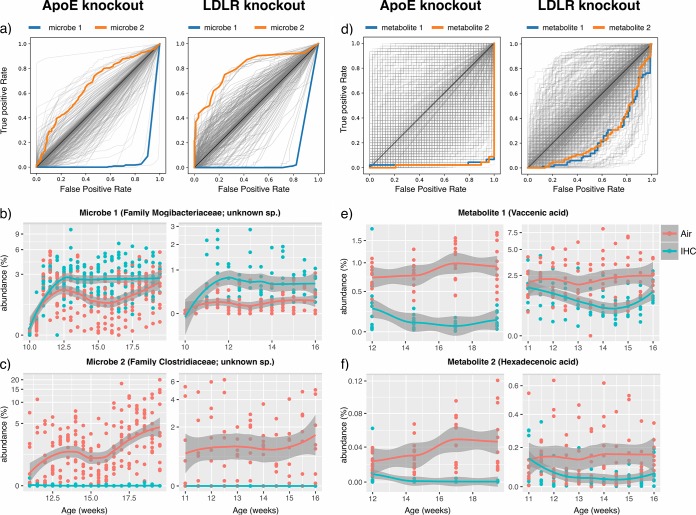
Individual microbes and metabolites that distinguish IHH from the control group in both *ApoE*^−/−^ and *Ldlr*^−/−^ mice. (a) ROC curves using each microbe’s abundance. Each curve represents the sensitivity and specificity as a function of the abundance of a single microbe to distinguish IHH and control groups. The curves for microbes enriched in IHH are above the diagonal line, while those for microbes depleted in IHH are below the diagonal line. Two predictive microbial features that are consistently altered in *ApoE*^−/−^ and *Ldlr*^−/−^ animals in both mouse models are highlighted by color. (b and c) The abundance trends of these two microbes in each mouse model along time. (d, e, and f) Similar plots for the metabolome data set highlighting two consistently altered features. The features were identified as vaccenic acid (*m*/*z*, 283.2629498309951; RT, 5.430859365079369) and hexadecenoic acid (*m*/*z*, 255.2317783582418; RT, 5.1814566985645945) based on MS/MS fragmentation. ROC, receiver operating characteristic; IHH, intermittent hypoxia and hypercapnia; RT, retention time.

The subsequent goal of this analysis was to narrow the list of fecal biomarkers that are reproducibly predictive of IHH exposure, thereby guiding future mechanistic and clinical studies. The RF classifiers used to distinguish the IHH-exposed and control animals described above provided us with a ranked list of bacterial and chemical features important for prediction (see the classifier trained on ApoE^−/−^ microbiome and metabolome data in Table S3 and the classifier trained on Ldlr^−/−^ data in Table S4 in the supplemental material). We examined the features that were top-ranked predictors in both Ldlr- and ApoE-based classifiers. To investigate if there were some key biomarkers that could single-handedly distinguish IHH from control, we used the abundance of each of these features individually to plot ROC curves and compute AUCs. Indeed, some of these microbial (Fig. 3a) and chemical (Fig. 3d) features could alone detect IHH exposure within each mouse model highly accurately (AUC > 0.75; see Tables S5 and S6 in the supplemental material for the AUC values per model per microbial and chemical feature, respectively). We then used our longitudinal data to compare trends of these predictive features in IHH-exposed and control groups in both animal models. Fig. 3b, c, e, and f show abundance trends in top consistently altered features (additional trends provided in Fig. S3 and S4 in the supplemental material). The goal was to investigate if these microbial and chemical species changed in the same direction on IHH exposure in both ApoE^−/−^ and Ldlr^−/−^ mice or had idiosyncratic responses to exposure based on the genetic background of the host. The consistent predictors included bacterial strains from the families *Mogibacteriaceae* and *Clostridiaceae*, fatty acids identified as vaccenic acid and hexadecenoic acid (level 2 identification [25]), and bile acids, including taurocholic acid, taurodeoxycholic acid, and muricholic acid (level 1 identification). These microbes and metabolites highlight key IHH-related changes in the gut microenvironment that could guide subsequent reconstitution experiments in germfree mice to establish causality.

10.1128/mSystems.00058-19.3 FIG S3 Additional metabolites that are top features distinguishing the IHH and control groups in both mouse models. (a and b) Heat maps showing the differentially abundant metabolomic features in *ApoE*^−/−^ and *Ldlr*^−/−^ mouse models. The *x* axis shows samples sorted by genotype, treatment, and age (weeks), and the *y* axis represents individual metabolites labeled using level 1 identification when possible or otherwise by *m*/*z* and retention time (RT_*m/z*). (a) Features of differential abundance between air and IHH groups in *ApoE*^−/−^ mice, with their abundances in *Ldlr*^−/−^ mice shown on the right part of the heat map. The green and red color bars on the right indicate whether the direction of change (decrease or increase in IHH) is the same or opposite in the two animal models (green, same direction; red, opposite direction; white, the feature that does not differ in the 2 groups in *Ldlr*^−/−^ model). (b) Similar heat map for the features of differential abundance between the air and IHH groups in the *Ldlr*^−/−^ model. (c to t) The plots are similar to Fig. 3e and f, showing the metabolite abundance trends along time. For trend plots, we used level 1 or 2 identification wherever annotation was possible. In other cases, we label the metabolite by *m*/*z* and retention time (*m/z*_RT). AUC, area under the ROC curve. Download FIG S3, DOCX file, 1 MB. Copyright © 2019 Tripathi et al. 2019 Tripathi et al. This content is distributed under the terms of the Creative Commons Attribution 4.0 International license.

10.1128/mSystems.00058-19.4 FIG S4 Additional microbes that are top features distinguishing IHH and control groups in both mouse models. (a and b) Heat maps showing the differentially abundant microbiome features in *ApoE*^−/−^ and *Ldlr*^−/−^ mouse models. The *x* axis shows samples sorted by genotype, treatment, and age (weeks), and the *y* axis represents individual microbes labeled by the highest taxonomic classification. (a) Features of differential abundance between the air and IHH groups in *ApoE*^−/−^ mice, with their abundances in *Ldlr*^−/−^ mice shown on the right part of the heat map. The green and red color bars on the right indicate whether the direction of change (decrease or increase in IHH) is the same or opposite in the two animal models (green, same direction; red, opposite direction; white, the feature that does not differ in the 2 groups in *Ldlr*^−/−^ model). (b) Similar heat map for the features of differential abundance between air and IHH groups in *Ldlr*^−/−^ model. (c to i) The plots are similar to Fig. 3b and c, showing the microbial abundance trends along time. AUC, area under the ROC curve. Download FIG S4, DOCX file, 1.5 MB. Copyright © 2019 Tripathi et al. 2019 Tripathi et al. This content is distributed under the terms of the Creative Commons Attribution 4.0 International license.

10.1128/mSystems.00058-19.7 TABLE S3 List of 16S amplicon features (a) and metabolites (b) ranked by their importance score in discriminating intermittent hypoxia and hypercapnia (IHH) exposure from controls in the *ApoE*^−/−^ mouse model. Download Table S3, XLS file, 0.2 MB. Copyright © 2019 Tripathi et al. 2019 Tripathi et al. This content is distributed under the terms of the Creative Commons Attribution 4.0 International license.

10.1128/mSystems.00058-19.8 TABLE S4 List of 16S amplicon features (a) and metabolites (b) ranked by their importance score in discriminating intermittent hypoxia and hypercapnia (IHH) exposure from controls in *Ldlr*^−/−^ mouse model. Download Table S4, XLS file, 0.2 MB. Copyright © 2019 Tripathi et al. 2019 Tripathi et al. This content is distributed under the terms of the Creative Commons Attribution 4.0 International license.

10.1128/mSystems.00058-19.9 TABLE S5 The AUCs using each microbial feature’s abundance to distinguish intermittent hypoxia and hypercapnia (IHH) exposure from controls in *ApoE*^−/−^ and *Ldlr*^−/−^ animals. AUC, area under the receiver operating characteristic curve. Download Table S5, XLS file, 0.7 MB. Copyright © 2019 Tripathi et al. 2019 Tripathi et al. This content is distributed under the terms of the Creative Commons Attribution 4.0 International license.

10.1128/mSystems.00058-19.10 TABLE S6 The AUCs using each metabolite feature’s abundance to distinguish intermittent hypoxia and hypercapnia (IHH) exposure from controls in *ApoE*^−/−^ and *Ldlr*^−/−^ animals. AUC, area under the receiver operating characteristic curve. Download Table S6, XLS file, 0.1 MB. Copyright © 2019 Tripathi et al. 2019 Tripathi et al. This content is distributed under the terms of the Creative Commons Attribution 4.0 International license.

## DISCUSSION

We examined the reproducibility of IHH-associated alterations in the gut microbiome and metabolome of Ldlr^−/−^ and ApoE^−/−^ mouse models, crucial for understanding links between OSA and associated cardiovascular pathologies. As both APOE and LDLR are important in clearing cholesterol and triglyceride-rich particles from the blood, both models show elevated plasma cholesterol levels. However, they develop atherosclerotic plaques to different extents under high-fat dietary conditions (26–28). Concordant with these phenotypic differences, we highlight throughout that the gut ecosystems of the two models are also intrinsically distinct. As technical variables such as origin of animals, housing conditions, experimental batches, and data acquisition protocols are important considerations for meta-analyses such as ours (29, 30), we ensured that all animals were handled in the same facility and data were acquired using identical protocols to minimize confounding effects. It is worth noting that instead of cohousing, we use separate cages for IHH-exposed and control animals (2 or 3 cages per treatment group) throughout, as previous work has shown that coprophagy during cohousing could rescue microbial and metabolic perturbations in the gut (31), which were of interest here. Furthermore, we used supervised machine learning to identify features specifically associated with IHH exposure in both animal models reproducibly.

To our knowledge, the impact of IHH on the gut ecosystem in the context of atherosclerosis has not been investigated before, making our work exploratory in nature. Intermittent hypoxia alone (without hypercapnia or HFD) has been reported to significantly alter the microbiome in wild-type mice (32) and guinea pigs (33), which lends support to our findings with IHH exposure. Another study modeled human OSA and its cardiovascular consequences in HFD-fed rats by inflating a tracheal balloon during the sleep cycle (34). The authors concluded that HFD and OSA synergistically caused hypertension and gut dysbiosis in these rats. Moreover, intermittent hypoxia with hypobaric stress (35) and chronic hypoxia (36) were also reported to alter the fecal microbiota in rats. In the latter study, chronic hypoxia-induced gut dysbiosis was implicated in premature senescence of bone marrow mesenchymal stem cells (BMSCs), and BMSCs were restored by intragastric supplementation of *Lactobacillus*. Interestingly, bone marrow function was also altered due to HFD-induced changes in the gut microbiota in mice (37). Further investigations would be needed to test if related mechanisms are at play in the obstructive sleep apnea mouse models discussed here (HFD and IHH exposure) as well.

In this work, we report consistent IHH-associated changes that include unclassified strains belonging to the families *Ruminococcaceae*, *Mogibacteriaceae*, *Lachnospiraceae*, and *Clostridiaceae* (Fig. 3; Fig. S4). These taxonomic groups have been associated with cardiovascular, metabolic, and inflammatory conditions (38–40), which indicates shared mechanistic pathways in OSA-associated cardiovascular conditions. Furthermore, our work is the first to profile OSA-associated changes in the gut metabolome at this scale. We observed reproducible perturbations in clinically relevant biomolecules in both ApoE^−/−^ and Ldlr^−/−^ mice. For example, vaccenic acid, a trans-fatty acid that has been reported to lower low-density lipoprotein (LDL) cholesterol and triglyceride levels in rats (41), was found to decrease under IHH exposure in both models. Similarly, bile acid molecules such as muricholic acid and taurocholic acid were more abundant in IHH-exposed versus control animals. Bile acids are crucial not only for facilitating transport of dietary fats and cholesterol in the host but also for regulating host energy expenditure, glucose homeostasis, and anti-inflammatory immune responses (42–46). Many metabolic and cardiovascular conditions (47) have been associated with aberrant bile acid profiles, suggesting that prolonged perturbations in these key molecules could contribute to downstream adverse cardiovascular consequences of OSA as well. It is noteworthy that we also identified microbes and metabolites that were highly predictive within both ApoE^−/−^ and Ldlr^−/−^ mice but altered in opposite directions in the two animals on IHH exposure (Fig. S3 and S4). Whether these opposite trends are due to a differential impact of HFD or IHH exposure on the two genotypes requires further investigation. This, together with the high cross-genotype prediction accuracy using all features (Fig. 2), suggests that although the microbiome and metabolome changes induced by IHH are largely consistent across mouse models, there do exist some animal model-specific changes as well. Hence, multi-animal model studies such as this are highly advantageous in precisely identifying biomarkers robustly associated with an intervention of interest.

In summary, our work provides reproducible candidate biomarkers of IHH exposure in animal models (and potentially OSA in humans) that will be most applicable to designing diagnostic and treatment modalities. Furthermore, we outline a general pipeline to select for biomarkers and therapeutic targets that is applicable to other intervention studies as well. We have made these information-rich data sets publicly available to promote collaborative progress in this area of research.

## MATERIALS AND METHODS

### Animals.

Atherosclerosis-prone 10-week-old male Ldlr^−/−^ (*n* = 16) and ApoE^−/−^ (*n* = 24) mice on a C57BL/6J background (stock no. 002207 and 002052, respectively; The Jackson Laboratory, Bar Harbor, ME) were used in this study (26, 27). Ldlr and ApoE deficiencies were confirmed by PCR according to the vendor's instructions. Animals were either exposed to intermittent hypoxia and hypercapnia (*n* = 8 and *n* = 12 for Ldlr^−/−^ and ApoE^−/−^ animals, respectively) or air (control group) and fed with high-fat diet. All animal protocols were approved by the Animal Care Committee of the University of California—San Diego and followed the *Guide for the Care and Use of Laboratory Animals* (48) of the National Institutes of Health.

### High-fat diet treatment.

Mice were fed with regular chow consisting of 0.01% cholesterol and 4.4% fat (TD.8604; Envigo-Teklad, Madison, WI) until initiation of dietary and IHH treatments. Starting at 10 weeks of age, male mice were provided with a high-fat diet (HFD) containing 1.25% cholesterol and 21% milk fat (4.5 kcal/g [TD.96121; Envigo-Teklad, Madison, WI]) while being exposed to either IHH or room air. Body weight of each mouse was measured twice a week. Food intake of animals in each cage was recorded twice a week.

### Intermittent hypoxia and hypercapnia exposure.

Intermittent hypoxia and hypercapnia (IHH) was maintained in a computer-controlled atmosphere chamber system (OxyCycler; Reming Bioinstruments, Redfield, NY) as previously described (4). IHH exposure was introduced to the mice in short periods (∼4 min) of synchronized reduction of O_2_ (from 21% to 8%) and increase of CO_2_ (from ∼0.5% to 8%) separated by alternating periods (∼4 min) of normoxia ([O_2_] = 21%) and normocapnia ([CO_2_] = ∼0.5%) with 1- to 2-min ramp intervals for 10 h per day during the light cycle. This treatment protocol mimics the severe clinical condition observed in obstructive sleep apnea patients. Mice on the same HFD but in room air were used as controls. Fecal samples were collected at baseline and twice each week for 6 weeks (Ldlr^−/−^) or 10 weeks (ApoE^−/−^).

### 16S rRNA sequence processing.

We performed 16S sequencing on fecal samples from Ldlr^−/−^ and ApoE^−/−^ mice for all time points. DNA extraction and 16S rRNA amplicon sequencing were done using Earth Microbiome Project (EMP) standard protocols (http://www.earthmicrobiome.org/protocols-and-standards/16s) (49). In brief, DNA was extracted using the Mo Bio PowerSoil DNA extraction kit (Carlsbad, CA). Amplicon PCR was performed on the V4 region of the 16S rRNA gene (Platinum Hot Start PCR 2× master mix; Invitrogen RED 13000014) using the primer pair 515f to 806r with Golay error-correcting barcodes on the reverse primer. Amplicons were barcoded and pooled in equal concentrations for sequencing. The amplicon pool was purified with the Mo Bio UltraClean PCR cleanup kit and sequenced on the Illumina HiSeq 2500 sequencing platform. Sequence data were demultiplexed and minimally quality filtered using the QIIME 1.9.1 script split_libraries_fastq.py, with a Phred quality threshold of 3 and default parameters to generate per-study FASTA sequence files.

The raw sequence data were processed using the Deblur workflow (23) with default parameters in Qiita (50). This generated a sub-operational taxonomic unit (sOTU) abundance per sample (BIOM format) (51). Taxonomies for sOTUs were assigned using the sklearn-based taxonomy classifier trained on the Greengenes 13_8 99% OTUs (feature classifier plug-in) in QIIME 2 (52). The sOTU table was rarefied to a depth of 2,000 sequences/sample to control for sequencing effort (53). A phylogeny was inferred using SATé-enabled phylogenetic placement (54), which was used to insert 16S Deblur sOTUs into Greengenes 13_8 at a 99% phylogeny.

### LC-MS/MS data processing.

We acquired LC-MS/MS data on fecal samples from Ldlr^−/−^ (for 10 through 16 weeks of age) and ApoE^−/−^ (at ages 10, 12, 14.5, 17, and 19.5 weeks) mice using identical protocols. Details of data acquisition parameters are specified in reference 2. Briefly, fecal pellets (30 to 50 mg approximately) were extracted in 500 µl of 50:50 methanol-H_2_O solvent, followed by centrifugation to separate insoluble material. The extracts were dried completely by centrifugal evaporation (CentriVap centrifugal vacuum concentrator; Labconco, Kansas City, MO) and resuspended in 150 µl of methanol-H_2_O (1:1 vol/vol). After resuspension, the samples were analyzed on a Vanquish ultraperformance liquid chromatography (UPLC) system coupled to a Q Exactive orbital ion trap (Thermo Fisher Scientific, Bremen, Germany). A C_18_ core shell column (Kinetex column, 50 by 2 mm, 1.7-µm particle size, 100-Å pore size; Phenomenex, Torrance, CA) with a flow rate of 0.5 ml/min (solvent A, H_2_O–0.1% formic acid [FA]; solvent B, acetonitrile–0.1% FA) was used for chromatographic separation (2).

The raw data sets were converted to *m*/*z* extensible markup language (mzXML) in centroid mode using MSConvert (part of ProteoWizard) (55, 56). All mzXML files were cropped with an *m*/*z* range of 75.00 to 1,000.00 Da. Feature extraction was performed in MZmine2 (http://mzmine.sourceforge.net/) (57) with a signal intensity threshold of 2.0e5 and minimum peak width of 0.3 s. The maximum allowed mass and retention time tolerances were 10 ppm and 10 s, respectively. A local minimum search algorithm with a minimum relative peak height of 1% was used for chromatographic deconvolution; the maximum peak width was set to 1 min. The detected peaks were aligned across all samples using the above-mentioned retention time and mass tolerances, producing the final feature table used in these analyses. (See the MZmine2 batch processing file available at https://github.com/knightlab-analyses/crossmodel_prediction/blob/master/data/metabolome/fileS7.mzmine2_batch.xml.)

We performed molecular networking (58, 59) in GNPS (https://gnps.ucsd.edu/) to putatively identify molecular features using MS/MS-based spectral library matches. The parameters used for molecular networking in this study are available at the University of California—San Diego GNPS site (https://gnps.ucsd.edu/ProteoSAFe/status.jsp?task=3dbc660b9bdd4f699d31750d99b25463). Additionally, we purchased analytical standards for bile acids of interest (based on previous work [2, 59]: α/β-muricholic acid, chenodeoxycholic acid, cholic acid, lithocholic acid, deoxycholic acid, and taurodeoxycholic acid) from Cayman Chemical (Ann Arbor, MI). We analyzed them using the same LC-MS/MS method described above to compare and verify the exact masses, fragmentation patterns, and retention times to ensure level 1 annotations (https://github.com/knightlab-analyses/haddad_osa/blob/master/data/standard_identified_all.txt), as defined by the 2007 metabolomics standards initiative (25).

### Sharedness of microbial and metabolomic features across animal models.

We calculated the sharedness of microbial features as follows. To quality control the 16S sequences obtained per animal model, we retained only reads that were prevalent within each model, i.e., above a sum relative abundance threshold of 10E−06 and present in at least 1% of the samples, thus avoiding sequencing noise. The numbers of such reads in Ldlr^−/−^ and ApoE^−/−^ animals were 635 and 582, respectively. Out of these, 248 sequences were shared between the two models. Therefore, the percentage of microbiome features shared between the animal models was 39% of unique microbial features found in the Ldlr^−/−^ model (and 42% of those in the ApoE^−/−^ model).

For metabolomic data, we quality controlled the chemical features by retaining those above a sum relative abundance threshold of 10E−01 and present in at least 10% of all samples for each animal model individually. There were 267 and 374 such features in Ldlr^−/−^ and ApoE^−/−^ animals, respectively. Out of these, 137 metabolites were shared between the two models. Thus, the percentages shared between the animal knockout models were 51% of total features in the Ldlr^−/−^ model and 36% of those in the ApoE^−/−^ model.

### Effect size analyses.

Effect sizes were calculated over the individual genotype, mice, cage number, age, and exposure type. For each of these covariates, we applied the mixed directional false-discovery rate (mdFDR) (60) methodology to test for the significance of each pairwise comparison among the groups. For each significant pairwise comparison via PERMANOVA (24), we computed the effect size using Cohen’s *d* (61) or the absolute difference between the mean of each group divided by the pooled standard deviation. As diversity estimators, we used unweighted UniFrac and Bray-Curtis distances matrices for the 16S rRNA sequencing and LC-MS/MS, respectively.

For the microbiome data layer (Table S1), when taking both genotypes together, we see that the first three largest effect sizes are mouse number, age, and cage number, followed by genotype and exposure type. It is noteworthy that the maximum difference (effect size) on the first three covariates are related to genotype differences. For example, the maximum difference in mouse number is between two mice (mouse no. 105 [ApoE^−/−^] versus 32 [Ldlr^−/−^] [Fig. S1]) that belong to two different genotypes and exposure types. To untangle the effect of genotype, we stratified our data set by genotypes and calculated effect sizes of each of the covariates within each model. The effects of covariates are ranked differently within each model, hinting toward underlying differences in the characteristics of the microbial community. Nevertheless, the effect of exposure is ranked comparably across models. Similarly, we calculate effect sizes of the above-mentioned covariates for the metabolome data layer (Table S2). When taking both genotypes together, consistent with the microbiome results, mouse number, age, and cage number have the largest effect sizes, and the groups with the maximum effects belong to different genotypes (e.g., mouse no. 114 [ApoE^−/−^] versus 17 [Ldlr^−/−^]). We then stratified the data by genotype and observed that different covariates had distinct effects within each genotype. Interestingly, our analysis shows that unlike in Ldlr^−/−^ mice, individual variability was not significant in ApoE^−/−^ mice. It is important to note that given our study design, we are reporting the effect sizes of each variable individually without correcting for other covariates. For example, it is not possible to partial out the effect of the cage from the effect IHH exposure as we needed to house IHH-exposed and control animals separately throughout the experiments (Fig. S1). Another study design will be needed to accurately report the independent effect size of each variable.

### Supervised classification.

The random forest (RF) classifier was trained and evaluated with cross-validation for each mouse model, using microbial or chemical features as predictors. During cross-validation, all the samples from the same mouse appeared only in either training or validation data but not both to avoid overoptimistic cross-validation accuracy scores as a result of the classifier learning idiosyncrasies of the individual itself rather than the treatment. The classifiers trained for each mouse model were then applied on the samples of the other mouse model for cross-genotype prediction. For the longitudinal prediction, we trained and evaluated an RF classifier on the samples collected at each time point for AUC computation. To assess the capability of individual 16S sequences and metabolites to separate IHH-exposed from control animals, we used the abundance of each feature as the score to plot the ROC curve and compute the AUC and highlighted the features that can single-handedly distinguish IHH on ROC plots. These analyses were done using the scikit-learn Python package.

### Data availability.

The data generated in this study are available publicly in the GNPS/MassIVE repository under the following accession numbers: for metabolomics data, MSV000081482 (Ldlr knockout animal) at ftp://massive.ucsd.edu/MSV000081482, MSV000082813 (ApoE knockout animal) at ftp://massive.ucsd.edu/MSV000082813, and MSV000081853 (commercial standards) at ftp://massive.ucsd.edu/MSV000081853, and for microbiome data, ERP106495 (Ldlr knockout animals; EBI database) and ERP110592 (ApoE knockout animals). Data analysis has been documented in Jupyter notebooks available on GitHub (https://github.com/knightlab-analyses/crossmodel_prediction).
