# Supplementary figures and images for: Intermittent Hypoxia and Hypercapnia Reproducibly Change the Gut Microbiome and Metabolome across Rodent Model Systems
Source: mSystems. 2019 Apr 30;4(2):e00058-19. doi: 10.1128/mSystems.00058-19 (PMC6495231; doi:10.1128/mSystems.00058-19)

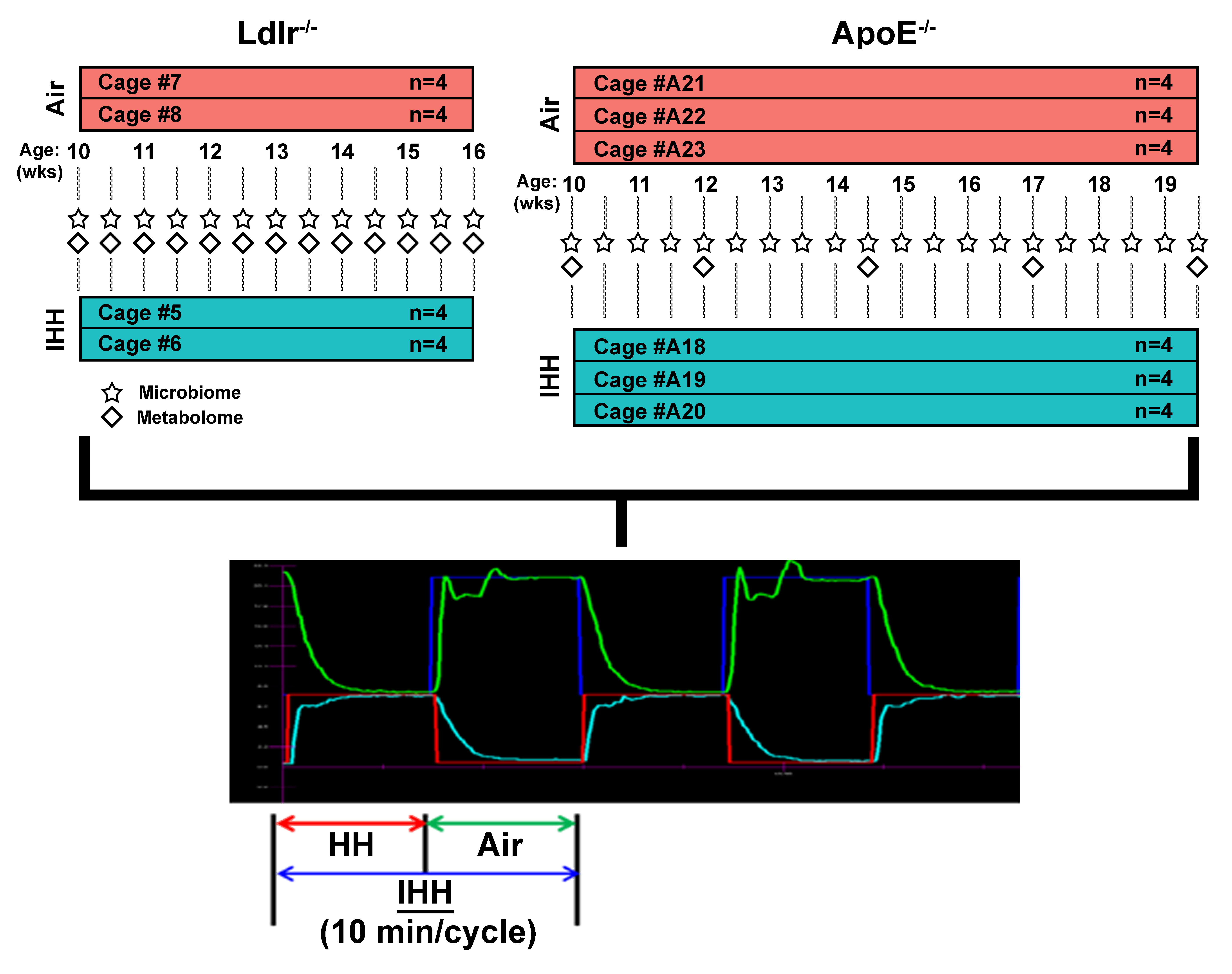

Supplement: FIG S1 [file mSystems.00058-19-sf001.tif]

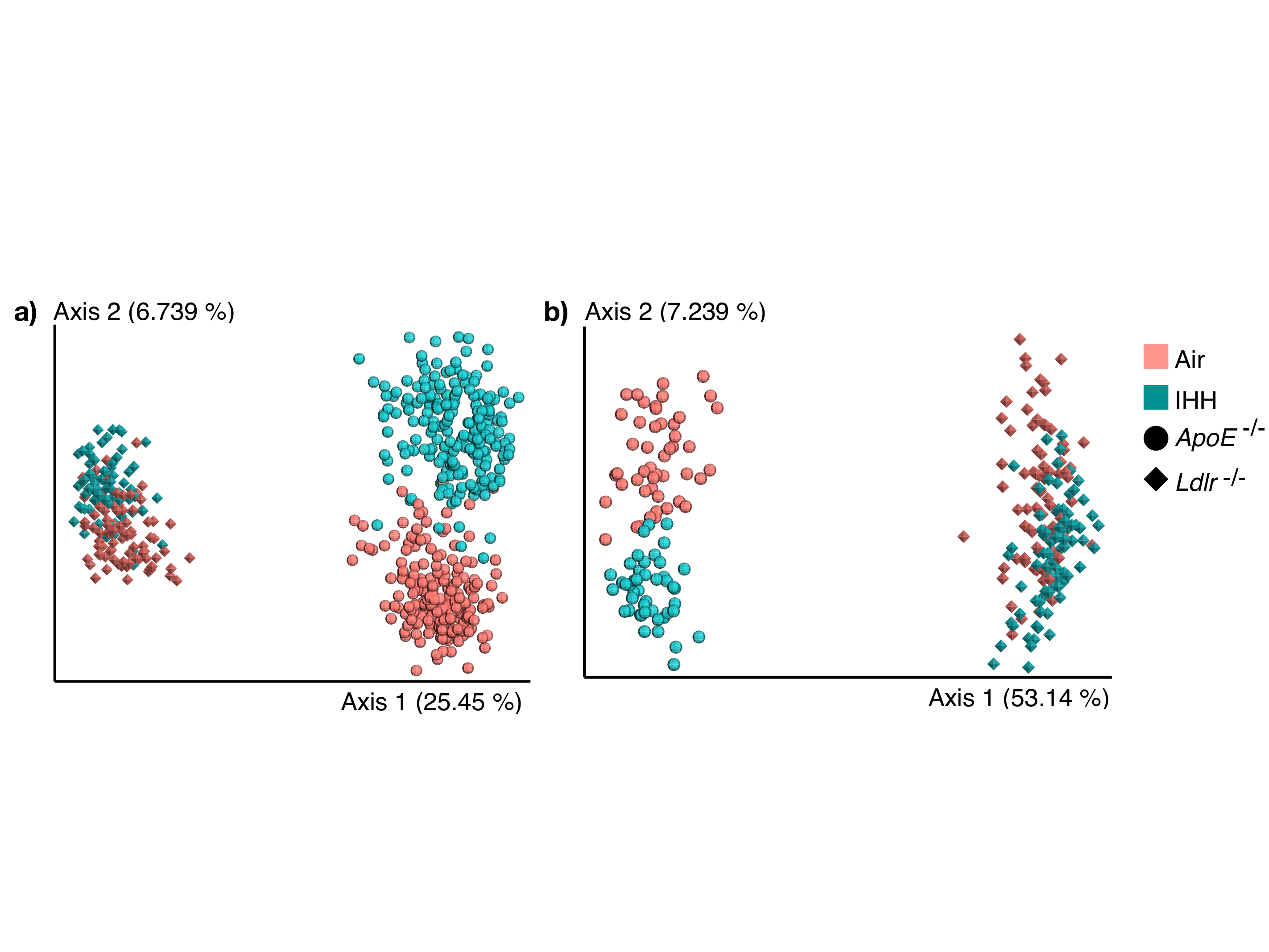

Supplement: FIG S2 [file mSystems.00058-19-sf002.tif]

**a)**

**b)**

**c)**

**d)**

**
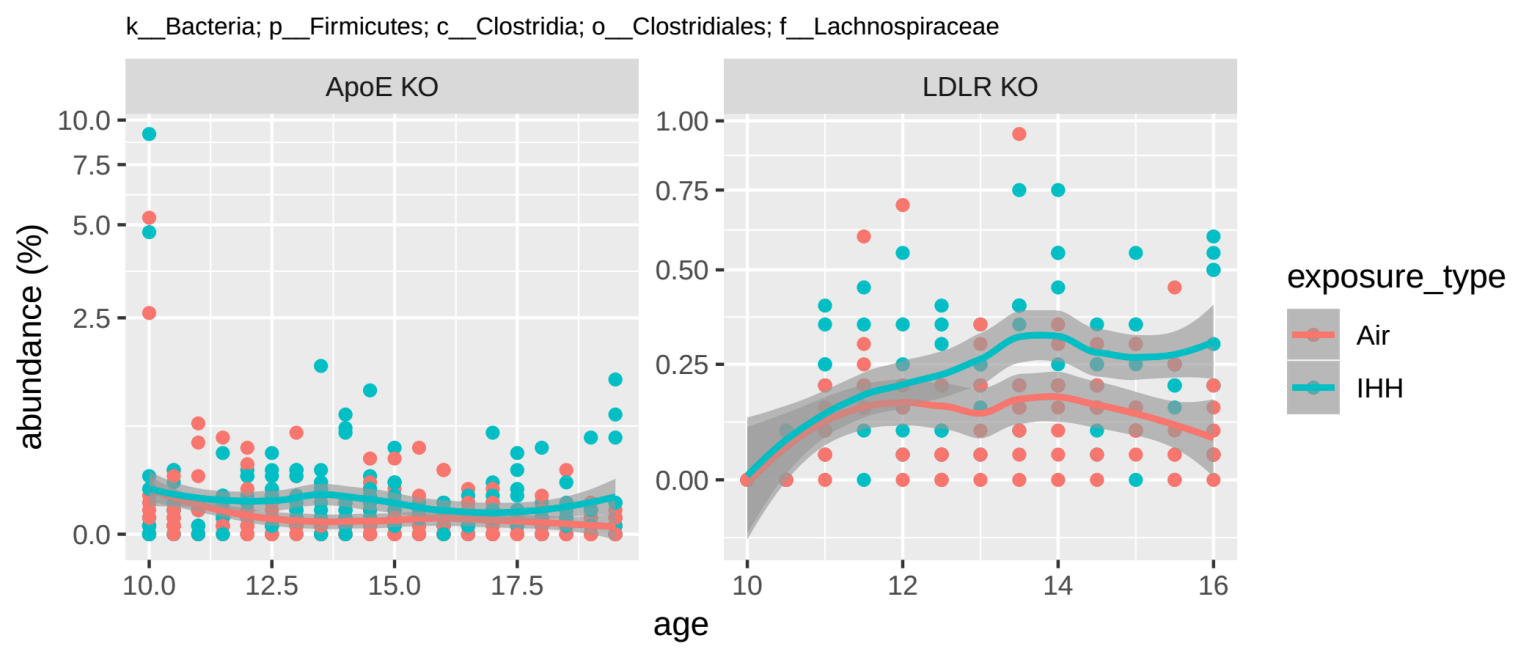
**

**e)**

**
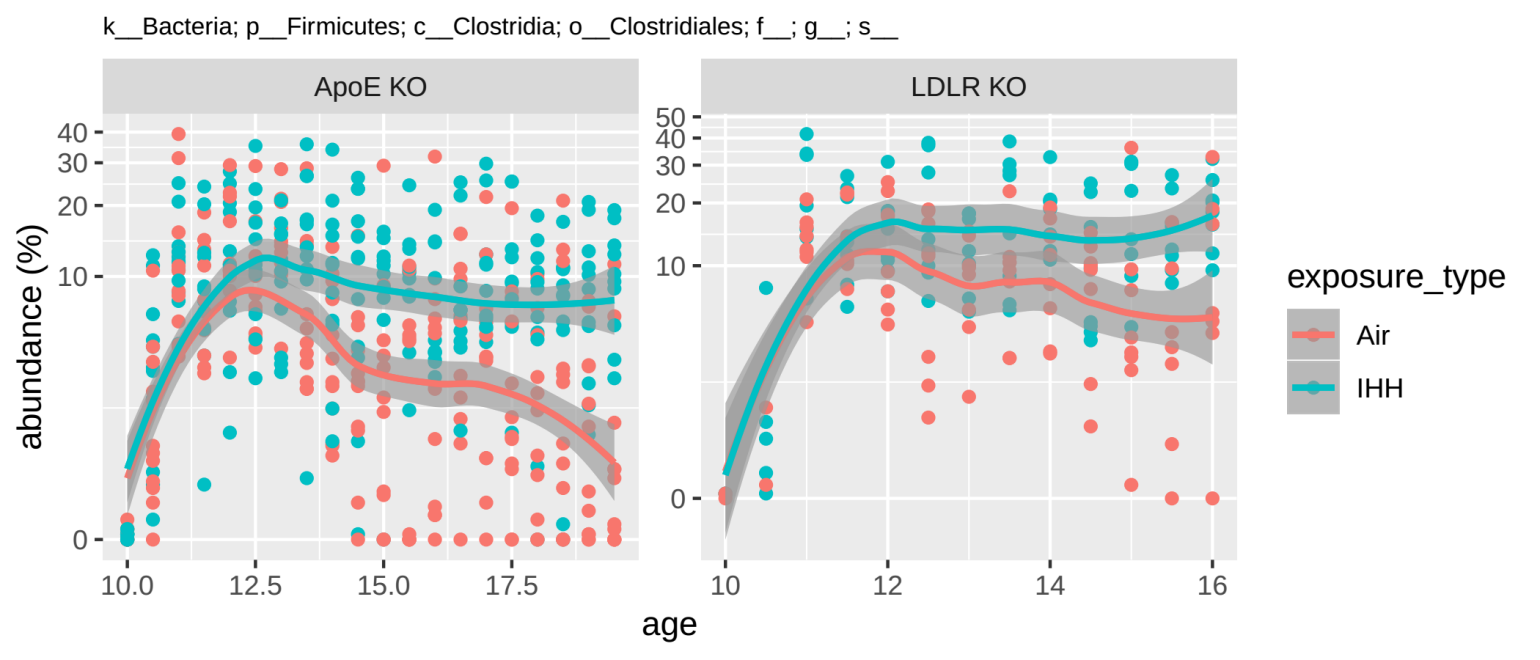
**

**f)
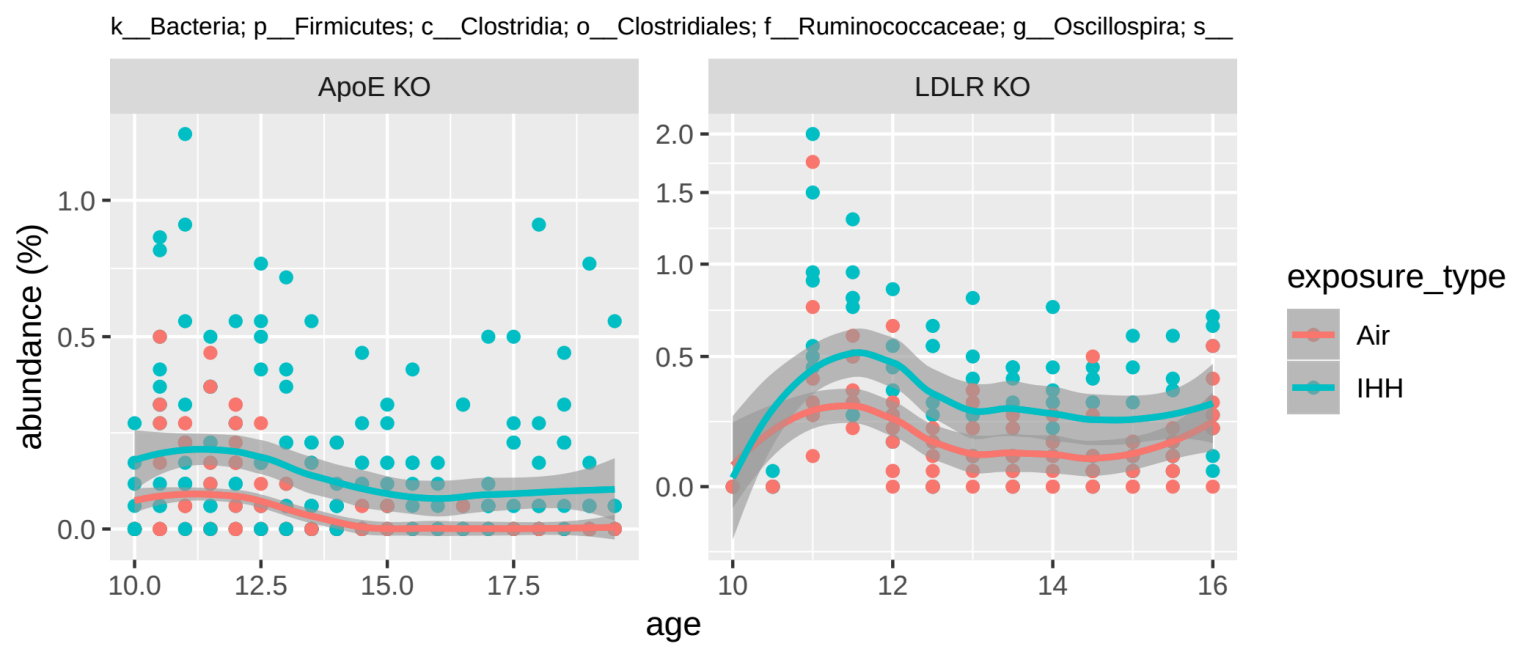
**

**g)
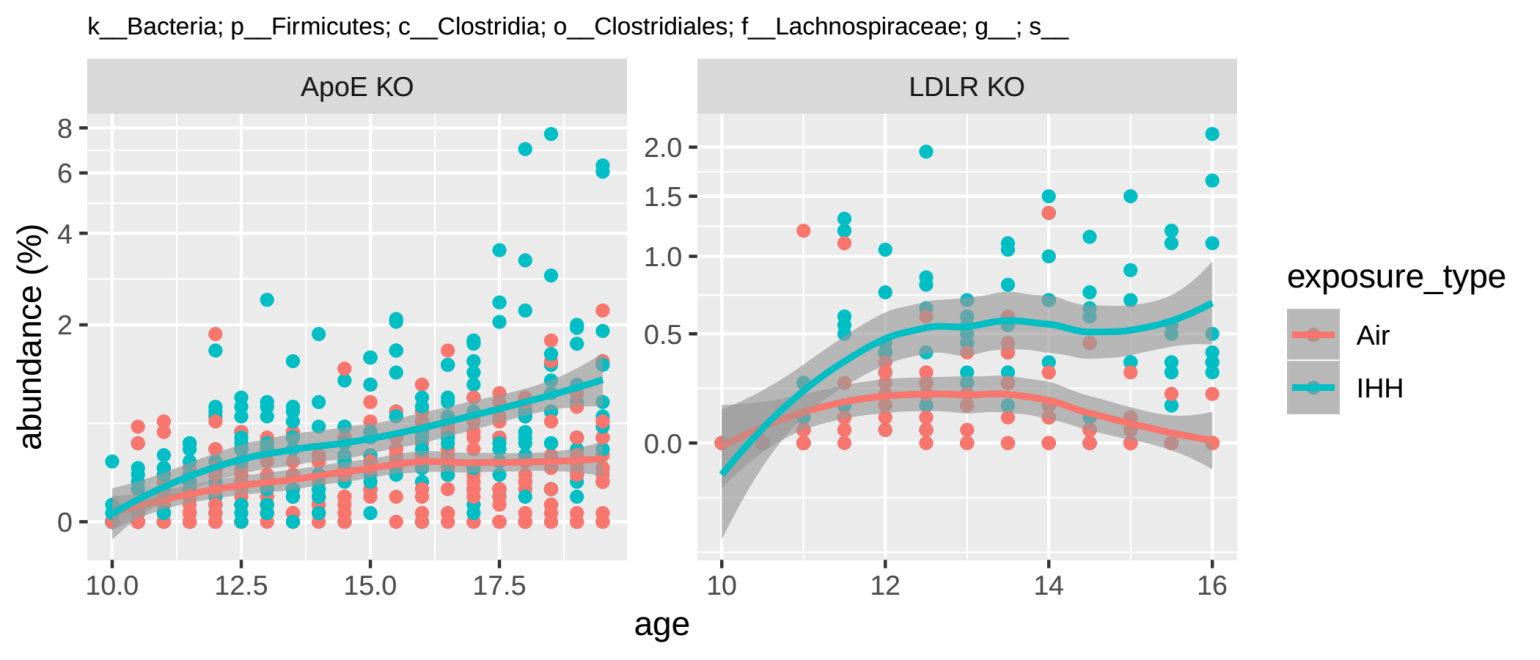
**

**h)
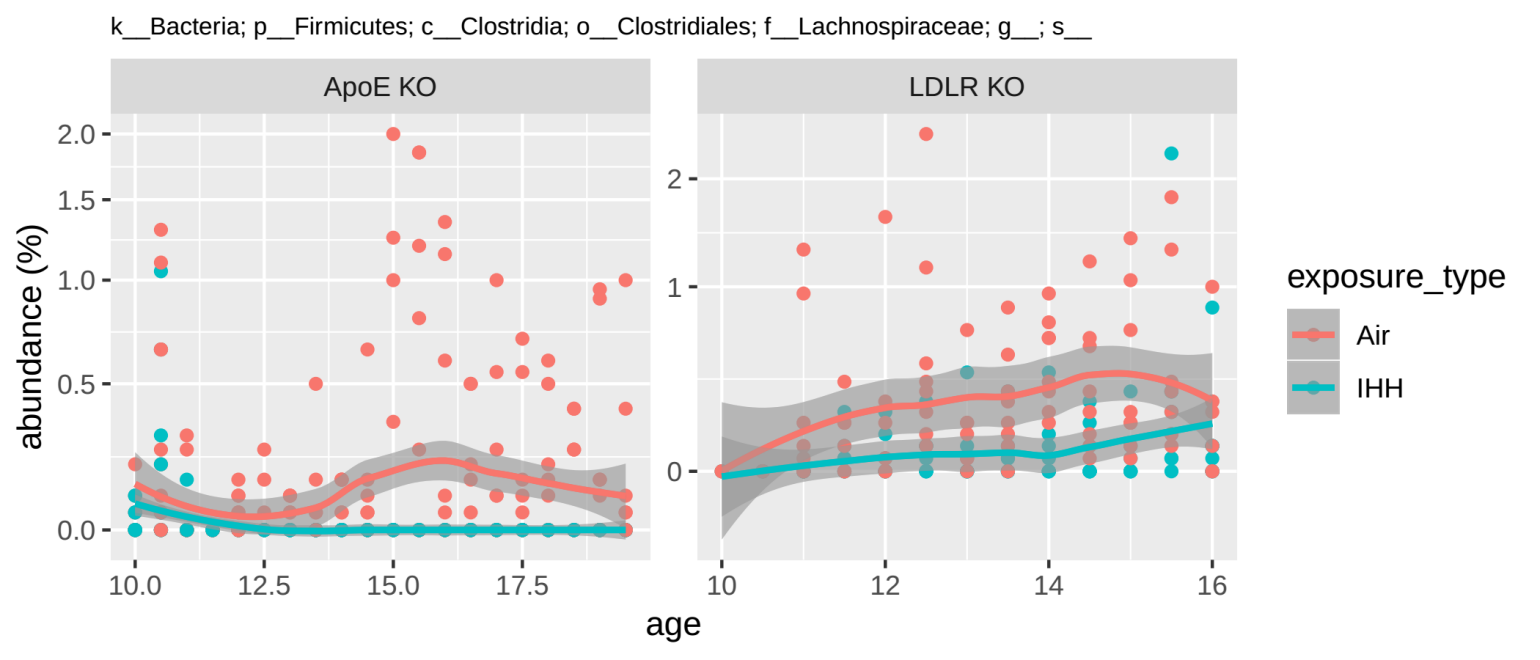
**

**i)**

**
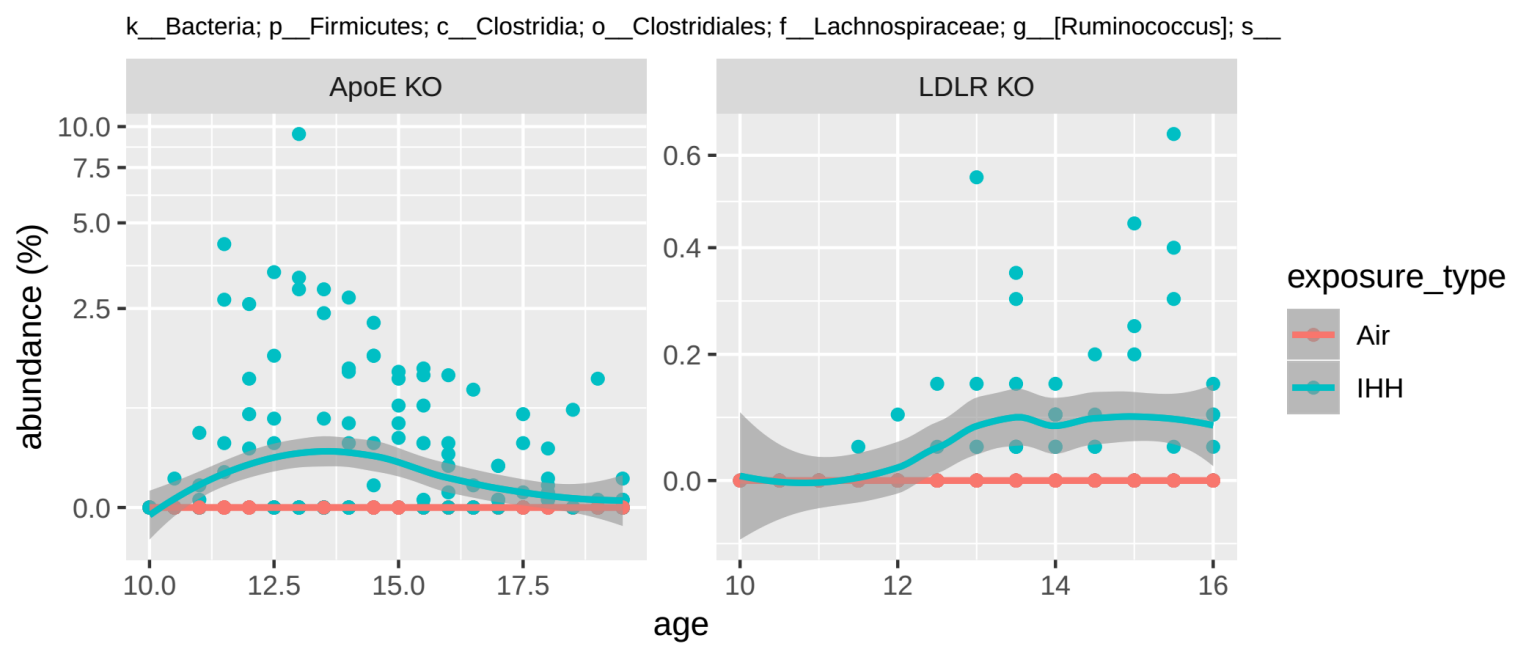
**

Supplement: FIG S4 [file mSystems.00058-19-sf004.docx]
