# Supplementary material for: Intermittent Hypoxia and Hypercapnia Reproducibly Change the Gut Microbiome and Metabolome across Rodent Model Systems
Source: mSystems. 2019 Apr 30;4(2):e00058-19. doi: 10.1128/mSystems.00058-19 (PMC6495231; doi:10.1128/mSystems.00058-19)
Supplement: TABLE S1 [file mSystems.00058-19-st001.docx]

**(a) Effect size for combined microbiome of both genotypes**

| **Covariates** | **Effect Size** | **Maximum difference** |
| --- | --- | --- |
| mouse_number | 5.09602958 | 105 vs. 32 |
| age | 2.658807628 | 11 vs. 17.5 |
| cage_number | 1.547058723 | A19 vs. 5 |
| genotype | 0.169107152 | LDLR knockout vs. ApoE KO |
| exposure_type | 0.039576453 | IHH vs. Air |

**(b) Effect size for Ldlr knockout mice microbiome**

| **Covariates** | **Effect Size** | **Maximum difference** |
| --- | --- | --- |
| mouse_number | 4.512154835 | 31 vs. 32 |
| cage_number | 0.936083608 | 5 vs. 6 |
| age | 0.194222924 | 11 vs. 14.5 |
| exposure_type | 0.032152194 | Air vs. IHH |

**(c) Effect size for ApoE knockout mice microbiome**

| **Covariates** | **Effect Size** | **Maximum difference** |
| --- | --- | --- |
| age | 2.59380138 | 17.5 vs. 11.5 |
| mouse_number | 1.738761074 | 117 vs. 104 |
| cage_number | 0.513379013 | A19 vs. A23 |
| exposure_type | 0.070543713 | Air vs. IHH |

**(d) Effect size for combined metabolome of both genotypes**

| **Covariates** | **Effect Size** | **Maximum difference** |
| --- | --- | --- |
| mouse_number | 4.512659741 | 114 vs. 17 |
| age | 1.993036059 | 11 vs. 11.5 |
| cage_number | 1.430865767 | 7 vs. A18 |
| genotype | 0.005828027 | ApoE KO vs. LDLR knockout |
| exposure_type | 0.003567181 | Air vs. IHH |

**(e) Effect size for Ldlr knockout mice metabolome**

| **Covariates** | **Effect Size** | **Maximum difference** |
| --- | --- | --- |
| mouse_number | 2.1406766 | 17 vs. 27 |
| age | 1.99303606 | 11 vs. 11.5 |
| cage_number | 0.53276647 | 7 vs. 5 |
| exposure_type | 0.19293636 | IHH vs. Air |

**(f) Effect size for ApoE knockout mice metabolome**

| **Covariates** | **Effect Size** | **Maximum difference** |
| --- | --- | --- |
| cage_number | 1.165264741 | A18 vs. A23 |
| exposure_type | 0.02531434 | IHH vs. Air |
| age | 0.01856508 | 12 vs. 17 |
| mouse_number | n.s | n.s |

n.s: not significant
