# Supplementary material for: Intermittent Hypoxia and Hypercapnia Reproducibly Change the Gut Microbiome and Metabolome across Rodent Model Systems
Source: mSystems. 2019 Apr 30;4(2):e00058-19. doi: 10.1128/mSystems.00058-19 (PMC6495231; doi:10.1128/mSystems.00058-19)
Supplement: TABLE S2 [file mSystems.00058-19-st002.docx]

**(a) microbiome**

| **age(weeks)** | **ApoE-/- AUC** |
| --- | --- |
| 10 | 0.875 |
| 10.5 | 0.909722 |
| 11 | 1 |
| 11.5 | 1 |
| 12 | 1 |
| 12.5 | 1 |
| 13 | 1 |
| 13.5 | 1 |
| 14 | 1 |
| 14.5 | 1 |
| 15 | 1 |
| 15.5 | 1 |
| 16 | 1 |
| 16.5 | 1 |
| 17 | 1 |
| 17.5 | 1 |
| 18 | 1 |
| 18.5 | 1 |
| 19 | 1 |

| **age(weeks)** | **Ldlr-/- AUC** |
| --- | --- |
| 11 | 0.714286 |
| 11.5 | 0.964286 |
| 12 | 0.78125 |
| 12.5 | 0.828125 |
| 13 | 0.875 |
| 13.5 | 0.890625 |
| 14 | 1 |
| 14.5 | 0.992188 |
| 15 | 1 |
| 15.5 | 1 |
| 16 | 0.991071 |

| **Age(weeks)** | **ApoE-/- AUC** |
| --- | --- |
| 12 | 0.875 |
| 14.5 | 0.909722 |
| 17 | 1 |
| 19.5 | 1 |

**(b) metabolome**

| **Age(weeks)** | **Ldlr-/- AUC** |
| --- | --- |
| 11 | 0.946429 |
| 11.5 | 1 |
| 12 | 0.890625 |
| 12.5 | 0.875 |
| 13 | 0.946429 |
| 13.5 | 0.96875 |
| 14 | 0.96875 |
| 14.5 | 1 |
| 15 | 1 |
| 15.5 | 0.964286 |
| 16 | 1 |
