# Supplementary material for: Intermittent Hypoxia and Hypercapnia Reproducibly Change the Gut Microbiome and Metabolome across Rodent Model Systems
Source: mSystems. 2019 Apr 30;4(2):e00058-19. doi: 10.1128/mSystems.00058-19 (PMC6495231; doi:10.1128/mSystems.00058-19)
Supplement: FIG S3 [file mSystems.00058-19-sf003.docx]

**a)**

**b)**

**c)**

**d)**

**e)**

**f)**

**g)**

**h)**

**i)**

**j)**

**k)**

**l)**

**m)**

**n)**

**o)**

**p)**

**q)**

**r)**

**s)**

**t)**
